# Dual Infection of a Pulmonary Hydatid Cyst by Aspergillus fumigatus in a Diabetic Adult: A Case Report From a Tertiary Care Hospital in the United Arab Emirates

**DOI:** 10.7759/cureus.90270

**Published:** 2025-08-17

**Authors:** Aqeel Saleem, Zaid Al Hassani, Mahmoud Al Deeb, Asad Khan, Ali Al Hassani

**Affiliations:** 1 Infectious Diseases, Sheikh Tahnoon Medical City, Al Ain, ARE; 2 College of Medicine, University of Sharjah, Sharjah, ARE

**Keywords:** aspergilloma, aspergillus fumigatus, echinococcus granulosus, immunocompetent, pulmonary hydatid cyst

## Abstract

Hydatid disease, caused by *Echinococcus granulosus*, most commonly affects the liver and lungs. Coinfection of a hydatid cyst with fungal organisms such as *Aspergillus fumigatus* is exceedingly rare, particularly in immunocompetent individuals. The aim of this case report is to describe the clinical course, diagnostic approach, and management of a patient with dual infection of a pulmonary hydatid cyst by *A. fumigatus*, highlighting its diagnostic challenges and implications for clinical practice.

We present a case of a 66-year-old male with type 2 diabetes mellitus, bronchial asthma, and chronic kidney disease, who developed a two-month history of productive cough, intermittent fever, and hemoptysis. Chest imaging revealed a cavitary lesion in the right upper lobe. Initial bronchoscopy and cultures identified *Klebsiella pneumoniae*, which was treated with intravenous antibiotics. Due to persistent symptoms, a second bronchoscopy revealed fungal hyphae and laminated cystic membranes, suggestive of coexisting aspergilloma and hydatid cyst. Despite treatment with oral voriconazole and albendazole, follow-up imaging demonstrated progression of the lesion. The patient subsequently underwent a right upper and middle lobectomy. Histopathological analysis confirmed dual infection with *Echinococcus granulosus* and *Aspergillus fumigatus*. He recovered uneventfully and remained disease-free at a three-year follow-up.

Early recognition of rare dual infections, such as a pulmonary hydatid cyst with *Aspergillus fumigatus* coinfection, is essential for improving outcomes. Clinicians should consider fungal coinfection when chronic pulmonary cavities fail to respond to empirical treatment, especially when bronchoscopy reveals fungal hyphae and laminated membranes. Researchers should further investigate the pathophysiology and optimal treatment strategies for such coinfections, while policymakers should ensure access to advanced diagnostic tools and multidisciplinary surgical care in endemic regions.

## Introduction

Cystic echinococcosis is a parasitic disease caused by *Echinococcus granulosus *and occurs in several endemic regions worldwide [1-4]. Although pulmonary involvement is recognized, fungal colonization of an intact hydatid cyst by *Aspergillus fumigatus *is exceptionally rare. This unusual presentation can closely resemble other chronic pulmonary conditions, creating significant diagnostic challenges. The present case report describes such a coinfection in a patient with chronic comorbidities, outlines the diagnostic approach, and discusses the clinical implications for management.

The liver is the most frequently involved organ, accounting for up to 80% of cases, followed by the lungs, which are affected in approximately 10% to 15% [5]. Pulmonary hydatid cysts may remain silent for years but can eventually cause symptoms such as cough, chest discomfort, hemoptysis, or signs of secondary infection as the cyst enlarges or ruptures [6].

Secondary infection of hydatid cysts, whether bacterial or fungal, is uncommon, especially in the lungs. Among fungal pathogens, *Aspergillus *species are opportunistic organisms that tend to colonize pre-existing pulmonary cavities in conditions such as tuberculosis, sarcoidosis, or malignancy, and are most often seen in immunocompromised patients [3,7]. The occurrence of *Aspergillus fumigatus* infection within an unruptured hydatid cyst in an immunocompetent individual is extremely rare, with only a few cases reported in the literature [8,9]. In this context, the term “immunocompetent” refers to the absence of underlying immunosuppressive disorders or treatments, such as malignancy, HIV infection, long-term corticosteroid therapy, or other causes of acquired or congenital immune deficiency.

This report presents a rare case of pulmonary coinfection with* Echinococcus granulosus* and *Aspergillus fumigatus* in an immunocompetent adult. It underscores the importance of considering fungal coinfection in chronic pulmonary cavities that do not respond to empirical treatment and highlights the role of multidisciplinary evaluation and timely surgical management in improving clinical outcomes.

## Case presentation

A 66-year-old male with a history of type 2 diabetes mellitus, hypertension, benign prostatic hyperplasia, diabetic retinopathy, stage III chronic kidney disease, and bronchial asthma presented with a two-month history of productive cough, intermittent fever, and hemoptysis. He denied weight loss, night sweats, recent travel, or use of immunosuppressive medications. On examination, he was alert and oriented. Chest auscultation revealed coarse crackles and diminished breath sounds over the right upper lung zone. Localized tenderness was noted on the right lateral chest wall. There was no digital clubbing or lymphadenopathy.

Laboratory evaluation showed leukocytosis, with a white blood cell count of 14.0 × 10⁹/L, accompanied by neutrophilic predominance at 78.8% and monocytosis at 14.1%. Hemoglobin was reduced at 116 g/L, with a mean corpuscular volume of 75 fL, consistent with microcytic anemia. Platelet count was elevated at 364 × 10⁹/L. Renal function was unchanged from baseline. HIV screening was non-reactive on two separate occasions, and hepatitis serologies were negative. *Aspergillus fumigatus*-specific IgE was undetectable (0.01 kIU/L), and there were no signs consistent with allergic bronchopulmonary aspergillosis. The complete results are summarized in Table 1.

**Table 1:** Initial laboratory investigations upon presentation.

Test	Result	Reference range
White blood cells (WBC)	14.0 × 10⁹/L	4.0 – 11.0 × 10⁹/L
Hemoglobin (Hgb)	116 g/L	120 – 160 g/L (F), 130 – 170 g/L (M)
Mean corpuscular volume (MCV)	75 fL	80 – 100 fL
Platelet count	364 × 10⁹/L	150 – 400 × 10⁹/L
Neutrophils	78.8%	40 – 70%
Monocytes	14.1%	2 – 10%
Sodium	132 mmol/L	135 – 145 mmol/L
Potassium	5.2 mmol/L	3.5 – 5.0 mmol/L
Chloride	103 mmol/L	98 – 107 mmol/L
CO₂ (bicarbonate)	21 mmol/L	22 – 29 mmol/L
Creatinine	114 µmol/L	62 – 115 µmol/L (M), 53 – 97 µmol/L (F)
Urea	6.1 mmol/L	2.5 – 7.1 mmol/L
Total protein	54 g/L	60 – 80 g/L
Albumin	24 g/L	35 – 50 g/L
Bilirubin (total)	8 µmol/L	3 – 21 µmol/L
Alkaline phosphatase (ALP)	61 IU/L	40 – 129 IU/L
Aspartate aminotransferase (AST)	28 IU/L	10 – 40 IU/L
Alanine aminotransferase (ALT)	12 IU/L	7 – 56 IU/L
C-reactive protein (CRP)	24 mg/L	<5 mg/L
Creatine kinase (CK)	517 IU/L	40 – 150 IU/L
Creatine kinase-myocardial band (CK-MB)	5.7 µg/L	0 – 5 µg/L
Troponin I	0.02 ng/mL	<0.04 ng/mL
HIV antigen/antibody screen	Nonreactive	Nonreactive
Hepatitis B surface antigen	Nonreactive	Nonreactive
Hepatitis B core antibody	Nonreactive	Nonreactive
Hepatitis C antibody	Nonreactive	Nonreactive
*Aspergillus fumigatus*-specific IgE	0.01 kIU/L	<0.35 kIU/L

**Figure 1:**
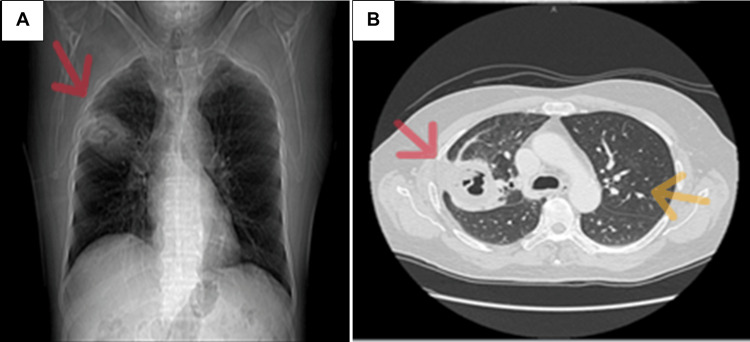
Initial chest radiograph and CT scan showing cavitary lesion and pulmonary nodules. (A) Chest X-ray demonstrating a right upper lobe cavitary opacity with associated volume loss. (B) Contrast-enhanced computed tomography (CT) of the thorax confirming a cavitary lesion in the right upper lobe measuring 5.3 × 4.8 cm with adjacent pleural reaction (red arrows). Also noted are a 6 mm subpleural nodule in the left lateral lung and a 4 mm nodule in the left upper lobe (yellow arrows).

On the second day of admission, a chest radiograph and contrast-enhanced computed tomography scan of the thorax revealed a thick-walled cavitary lesion in the right upper lobe measuring 5.3 x 4.8 centimeters. The lesion was associated with surrounding pleural thickening and parenchymal inflammation. Several small pulmonary nodules, ranging from 4 to 6 millimeters, were also seen in the left lung. These findings are illustrated in Figure 1.

On the fourth day of hospitalization, the patient underwent flexible bronchoscopy with transbronchial biopsy and bronchoalveolar lavage. Histopathologic examination of the biopsy revealed eosinophilic proteinaceous material containing fungal hyphae with acute angle branching, morphologically consistent with *Aspergillus *species. Special stains confirmed the presence of fungal elements. A small portion of laminated membranous tissue also stained positive with periodic acid-Schiff (PAS) and PAS-diastase, raising suspicion for a hydatid cyst membrane. Due to the limited nature of the sample, interpretation required correlation with clinical findings and imaging. There was no histological evidence of granulomatous inflammation or malignancy. Cultures from the bronchoalveolar lavage grew *Klebsiella pneumoniae*, and the patient was started on intravenous cefepime, 2 grams every 12 hours, for a total of 10 days.

Although the patient experienced temporary relief from fever and sputum production, his symptoms persisted. Approximately one month after the initial bronchoscopy, a repeat procedure was performed in light of an ongoing cough and abnormal imaging results. Endobronchial inspection revealed cystic structures, and subsequent biopsy once again demonstrated fungal elements along with more abundant laminated membranous material. Serologic testing for *Echinococcus *returned positive with a titer of 1 to 128, confirming the diagnosis of pulmonary hydatid disease with secondary fungal infection.

The patient was started on dual oral therapy consisting of voriconazole, 300 milligrams twice daily, and albendazole, 400 milligrams twice daily, for two months. Voriconazole was initially administered intravenously for three weeks before transitioning to oral therapy. He remained under regular outpatient follow-up for the following two months.

**Figure 2:**
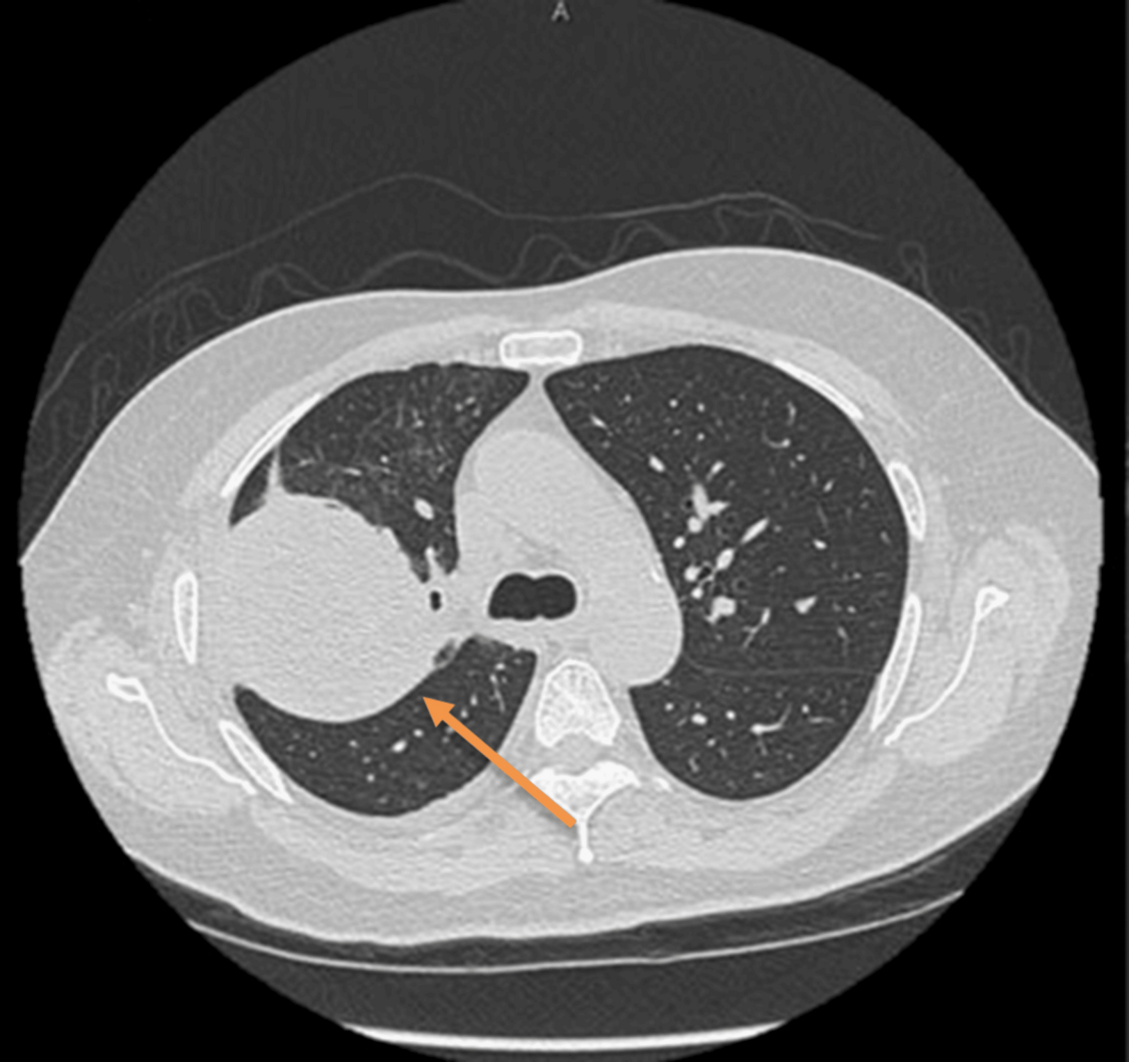
Thoracic CT imaging before lobectomy for pulmonary hydatid cyst with aspergilloma. Follow-up thoracic computed tomography (CT) scan performed two months after bronchoscopy showing interval progression of the right upper lobe cavitary lesion, now measuring 8 × 7 cm with multiple internal air loculi. Newly developed patchy parenchymal shadowing is seen in the apical segment of the right upper lobe. Trace right pleural effusion and reactive pleural thickening are also noted.

Despite this regimen, a follow-up thoracic computed tomography scan showed progression of the cavitary lesion, which had enlarged to 8 x 7 centimeters and contained multiple internal air loculi. Patchy parenchymal infiltrates had developed in the apical segment of the right upper lobe, accompanied by a trace pleural effusion and reactive pleural thickening. These imaging findings are presented in Figure 2. Given the lesion’s continued progression and failure to respond to medical management, surgical intervention was recommended.

The patient subsequently underwent a right posterolateral thoracotomy with resection of the upper and middle lobes and placement of two chest tubes. Intraoperatively, an 8 x 6 centimeter cystic cavity containing purulent fluid, necrotic debris, and laminated membranes was identified. Histopathologic examination of the resected specimen confirmed a hydatid cyst, with laminated membranes staining positive for PAS and PAS-diastase, diagnostic of* Echinococcus granulosus*. Numerous septate fungal hyphae, morphologically consistent with *Aspergillus *species, were found infiltrating the cyst wall. No malignant features were identified in the sampled lymph nodes. Abdominal ultrasonography ruled out hepatic hydatid involvement. Unfortunately, despite multiple attempts, the original histopathologic images could not be retrieved from the hospital’s electronic records and are therefore not included in this report.

Following surgery, the patient received an initial dose of caspofungin 70 milligrams intravenously, followed by 50 milligrams daily for seven days. Oral voriconazole and albendazole were continued throughout the hospitalization and beyond. The patient remained on voriconazole, 200 milligrams twice daily, and albendazole, 400 milligrams twice daily, for a total duration of one year after lobectomy.

**Figure 3:**
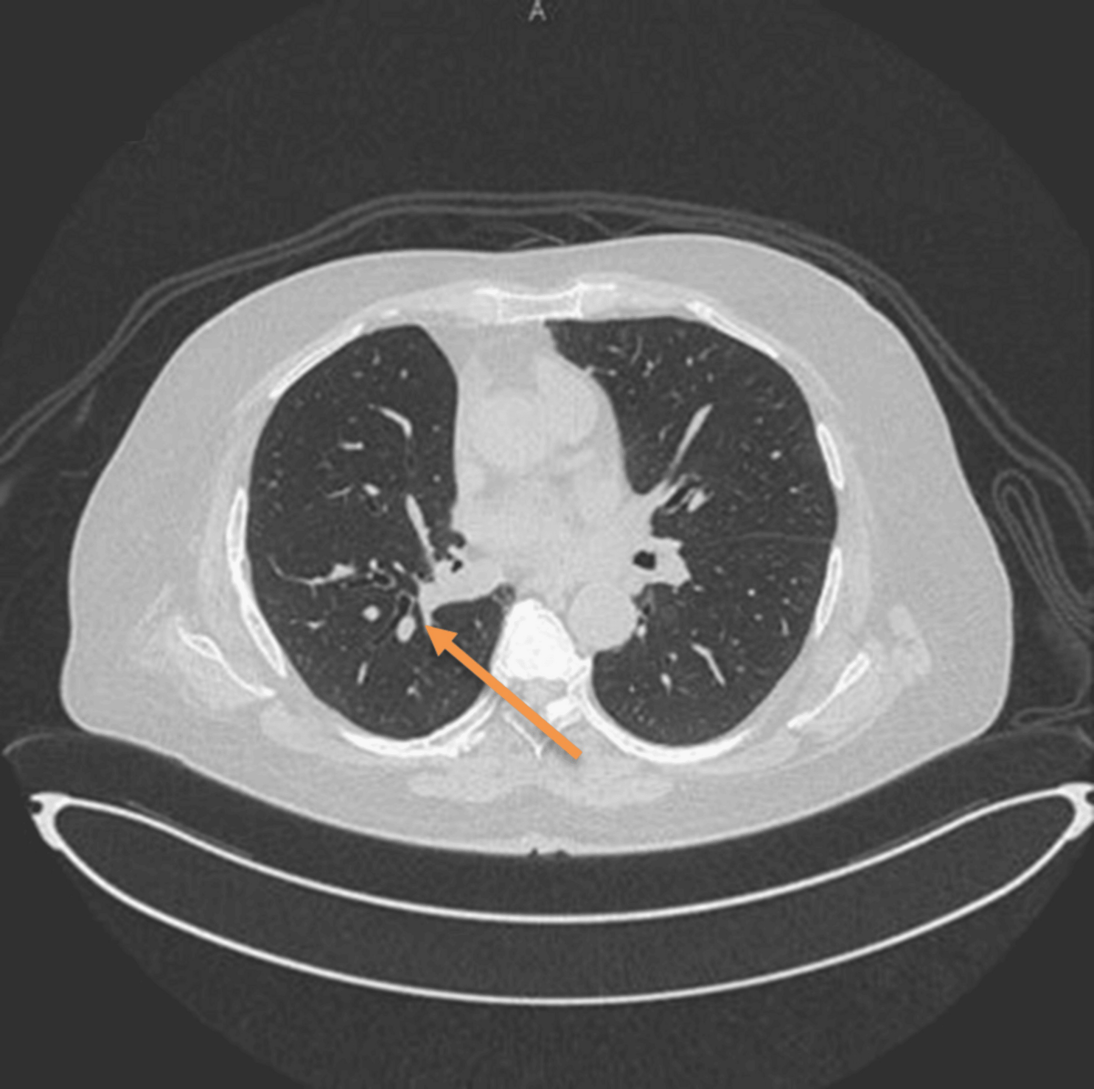
Thoracic CT imaging after lobectomy for pulmonary hydatid cyst with aspergilloma. Thoracic CT scan obtained one year after right upper and middle lobectomy demonstrating near-complete resolution of the prior cavitary lesion, with no evidence of residual disease or nodularity.

A follow-up thoracic computed tomography scan performed one year postoperatively demonstrated near-complete resolution of the previously identified cavitary lesion. Mild bronchiectatic and fibrotic changes were noted in the right lower lobe, but there was no evidence of residual nodules or mediastinal lymphadenopathy. These imaging findings are presented in Figure 3. The patient remained asymptomatic and functionally independent, with no recurrence detected during three years of outpatient follow-up.

## Discussion

Hydatid cysts, caused by *Echinococcus granulosus*, most frequently develop in the liver and lungs. Initially, patients with hydatid disease are usually asymptomatic. Symptoms typically arise only after cysts enlarge or develop complications, presenting as cough, chest pain, hemoptysis, or secondary infections, influenced by the cyst's size, location, and involvement of adjacent structures [2,6]. Consequently, pulmonary hydatid disease often remains undiagnosed until complications prompt medical evaluation.

Co-infection of pulmonary hydatid cysts with fungi such as *Aspergillus* species is uncommon, particularly among immunocompetent individuals [9,10]. Typically, aspergillomas occur after the rupture or surgical intervention of a hydatid cyst and are commonly associated with structural lung defects or compromised immunity [10,11]. In contrast, our patient exhibited a hydatid cyst without identifiable immune dysfunction, suggesting a less recognized mechanism of fungal colonization.

Given their infrequent occurrence, fungal infections within hydatid cysts remain poorly understood. Differentiating these cases from other cavitary pulmonary conditions is diagnostically challenging [10,12]. Clinical presentations frequently overlap, with patients typically exhibiting cough, hemoptysis, fever, and cavitary lesions on imaging [1,10]. Radiologically, hydatid cysts exhibiting internal fluid levels or partial evacuation, known as the "water lily sign," can resemble pulmonary abscesses, neoplasms, or fungal balls, complicating accurate diagnosis [13]. Therefore, definitive diagnosis commonly requires surgical exploration and histopathologic confirmation [3].

*Aspergillus fumigatus*, a saprophytic fungus, is known to cause various pulmonary conditions, including aspergilloma, semi-invasive disease, and invasive aspergillosis [1]. In the context of hydatid cysts, fungal colonization usually occurs after cyst rupture, surgical intervention, or in the presence of structural lung abnormalities [1,10]. Larger hydatid cysts may predispose patients to secondary infections by creating ideal environments for microbial growth, whereas cysts located near hilar structures can resist natural resolution and thus allow persistent fungal colonization [11]. Nevertheless, fungal infections have occasionally been reported without these classical risk factors [14].

Management of hydatid cysts complicated by *Aspergillus* co-infection is often challenging, especially in patients resistant to conventional medical therapy [15]. While antifungal and antiparasitic medications may be initiated empirically, surgical resection remains the definitive treatment for symptomatic or refractory cases [13,15,16]. Medical therapy with albendazole or mebendazole typically serves as adjunctive or alternative treatment in patients with unresectable, recurrent, or disseminated disease [15]. In our case, dual infection was definitively identified only after lobectomy, which revealed laminated hydatid membranes and fungal hyphae upon histopathologic examination.

Microscopic examination using special fungal stains, such as PAS and Gomori methenamine silver, is essential to confirm the diagnosis accurately [15,17]. The patient’s immunocompetent status likely limited fungal spread, enabling a favorable postoperative recovery.

Ultimately, this case highlights the importance of maintaining a high degree of suspicion for fungal co-infection in patients presenting with persistent pulmonary cavities, even when hydatid cysts appear intact and immunity appears uncompromised. In such scenarios, clinicians should carefully evaluate the possibility of uncommon infections, pursuing thorough diagnostic assessment with imaging, bronchoscopy, and surgical exploration when standard antibiotic treatment fails to yield improvement.

## Conclusions

Pulmonary hydatid cysts, though uncommon, are a notable cause of cavitary lung lesions. Superimposed infection with *Aspergillus *species is rare, particularly in individuals without underlying immunosuppression. This case underscores the clinical importance of co-infection with *Echinococcus granulosus* and *Aspergillus fumigatus*, which can closely resemble other chronic pulmonary diseases in both presentation and imaging. Establishing an accurate diagnosis requires a multimodal approach that includes radiological assessment, microbiological testing, and histopathological confirmation. When both infections are localized, surgical resection remains the definitive treatment. Clinicians should remain vigilant for potential fungal co-infection in patients with complicated hydatid disease, even in the absence of immunodeficiency, as early recognition is essential for guiding effective management and improving patient outcomes.
